# Clinical Impact of Prophylactic Antibiotic Treatment for Self-Expandable Metallic Stent Insertion in Patients with Malignant Colorectal Obstruction

**DOI:** 10.1155/2015/416142

**Published:** 2015-03-31

**Authors:** Jong-Sun Kim, Wan-Sik Lee, Cho-Yun Chung, Hyung-Chul Park, Dae-Seong Myung, Chan-Young Oak, Mi-Young Kim, Mi-Ok Jang, Seung-Ji Kang, Hee-Chang Jang, Sung-Bum Cho, Hyun-Soo Kim, Young-Eun Joo

**Affiliations:** ^1^Division of Gastroenterology, Department of Internal Medicine, Chonnam National University Medical School, Gwangju, Republic of Korea; ^2^Division of Infectious Diseases, Department of Internal Medicine, Chonnam National University Medical School, Gwangju, Republic of Korea

## Abstract

*Purpose*. The aim of this study was to determine the efficacy of prophylactic antibiotics (PA) for reducing the infectious complications and the potential risk factors responsible for the infectious complications after stent insertion for malignant colorectal obstruction. *Methods*. We performed a retrospective review of 224 patients who underwent self-expandable metallic stent (SEMS) insertion for malignant colorectal obstruction from May 2004 to December 2012. *Results*. There were 145 patients in the PA group and 79 in non-PA group. The CRP level in PA group was significantly higher than that in non-PA. Abdominal tenderness and mechanical ileus were significantly more frequent in PA group than those in non-PA. The frequency of post-SEMS insertion fever, systemic inflammatory response syndrome (SIRS), and bacteremia was not significantly different between PA and non-PA groups. In multivariate analysis, the CRP level was risk factor related to post-SEMS insertion SIRS. However, in propensity score matching analysis, there was no independent risk factor related to post-SEMS insertion fever, SIRS, and bacteremia. *Conclusion*. The use of PA in patients with malignant colorectal obstruction may be not effective to prevent the development of infectious complications after SEMS insertion.

## 1. Introduction

Colorectal cancer is one of the leading causes of cancer-associated morbidity and mortality in the world, and the incidence of colorectal cancer has been increasing rapidly, especially in Asia [1]. Up to 30% of patients with colorectal cancer can present with acute obstruction of large bowel at the time of diagnosis and require emergent colorectal surgery [2, 3]. Emergency colorectal surgery for acute obstruction is associated with an increased risk of morbidity and mortality in comparison with elective surgery.

Self-expandable metallic stent (SEMS) insertion has been known initially to be effective and safe for relief of malignant colorectal obstruction and has now gained acceptance, either as a palliative treatment or as a bridge to elective surgery [4–6]. Recently, SEMS insertion in malignant colorectal obstruction is recommended for patients with clinical symptoms and imaging evidence, as an alternative treatment to emergency surgery in patients with high risk of postoperative mortality and as palliative treatment according to the clinical guideline published by European Society of Gastrointestinal Endoscopy (ESGE) [7] and reviewed and endorsed by the Governing Board of American Society for Gastrointestinal Endoscopy (ASGE) [8]. However, SEMS insertion is not recommended as a bridge to elective surgery and prophylactic treatment [7, 8].

SEMS insertion has the potential to allow clean bowel preparation, clinical stabilization, and evaluation of the entire colon for synchronous lesions. However, a variety of complications after SEMS insertion can occur as follows; perforation, malposition, reobstruction, migration, bleeding, and infectious complications [9–11]. Although the incidence of endoscopic procedures-related infectious complications is rare, it remains one of the serious complications of endoscopic procedures when it occurs. The use of prophylactic antibiotics (PA) during high-risk endoscopic procedures is known to reduce the risk of significant endogenous infectious complications in a guideline published by the ASGE [12].

Colonoscopic procedure requires inflation of the bowel and can irritate the bowel wall during manipulation, which can enhance the transmural migration of intestinal microbial flora across the bowel wall and cause subsequent infectious complications. SEMS insertion may be prone to cause the infectious complications because of the increase of wall tension and mucosal trauma during procedure and after. Therefore, a significant number of clinicians recommend PA to prevent the infectious complications after stent insertion, especially when patients with colorectal cancer present with acute emergency obstruction [10]. Recently, the routine use of PA may be unnecessary before SEMS insertion because SEMS insertion did not induce significant bacteremia in colorectal obstruction [13]. However, the benefit of PA in preventing the infectious complications including fever, systemic inflammatory response syndrome (SIRS), bacteremia, and sepsis in patients receiving SEMS insertion is not well-documented. The aim of this study was to determine the efficacy of PA for reducing the infectious complications and the potential risk factors responsible for the infectious complications after SEMS insertion for malignant colorectal obstruction.

## 2. Methods

### 2.1. Patients

**Figure 1:**
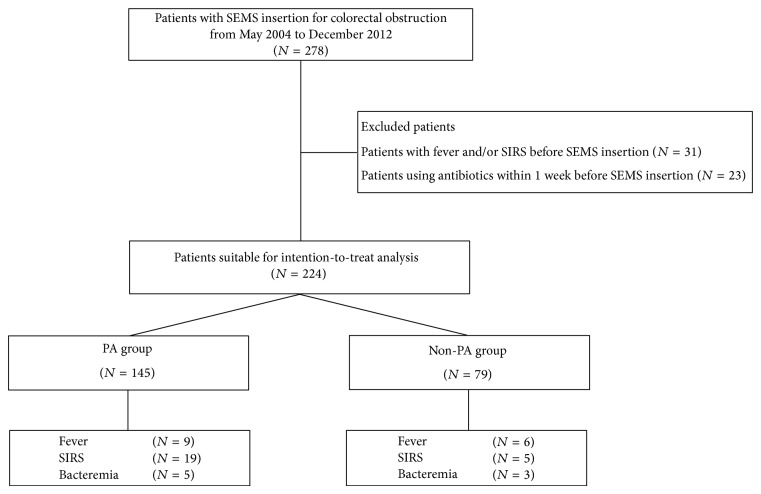
Flow chart showing patient selection of the allocation of malignant colorectal obstruction. SEMS, self-expandable metallic stent; *N*, number; PA, prophylactic antibiotic; SIRS, systemic inflammatory response syndrome.

Two hundred and seventy-eight patients who had received a SEMS for malignant colorectal obstruction from May 2004 to December 2012 at Chonnam National University Hwasun Hospital (Jeonnam, Korea) were analyzed retrospectively (Figure 1). Among them, 31 patients with fever (body temperature over 38.0°C) and/or SIRS and 23 patients taking antibiotics within 1 week before the insertion of SEMS were excluded. A total of 224 patients were finally enrolled. 145 of 224 patients received antibiotics before the insertion of SEMS (PA group) and the remaining 79 patients did not receive PA (non-PA group) (Figure 1). Third generation cephalosporin (ceftizoxime sodium or cefodizime sodium 2 gram two times per day, intravenous) and/or metronidazole (500 mg three times per day, intravenous) were used for prophylaxis at the time of admission before the insertion of SEMS. All the patients had overt clinical features of colorectal obstruction such as nausea, vomiting, abdominal pain, tenderness, abdominal distention, or failure to pass feces and gas. Colorectal obstruction was diagnosed clinically and radiologically. All the patients received abdominal X-ray, colonoscopy, and computed tomography (CT) to evaluate the stage of colorectal cancer and to check the site, degree, and length of obstruction before the insertion of SEMS. This study was approved by the Institutional Review Board of Chonnam National University Hwasun Hospital (IRB number: CNUHH-2012-28).

### 2.2. Clinical Protocol

SEMS insertion was principally indicated for malignant colorectal obstruction corresponding to the criteria based on obstructive symptoms, colonoscopic findings, and abdominal CT as described previously. Informed consent with adequate explanation of stent insertion and possible complications was obtained from each patient. Under the fluoroscopic guidance, colonoscope (CF-240 l; Olympus, Tokyo, Japan) or 2-channel therapeutic endoscope (GIF-2T240; Olympus, Tokyo, Japan) was inserted to the level of the obstruction. The obstruction was passed with an endoscopic retrograde cholangiopancreatography catheter (MTW Endoskopie, Wesel, Germany). After passing through the obstruction, the catheter was advanced over the 0.038-inch angled or straight stiff-type guidewire (Glidewire; Terumo, Tokyo, Japan) to the proximal region of obstruction. The guidewire was removed and a contrast dye (Gastrografin; Schering, Berlin, Germany) was injected to delineate the length, site, and morphology of obstruction. The catheter was then replaced by the guidewire. SEMS deliver catheter was advanced over the guidewire and positioned through the obstruction. Upon the release of SEMS delivery catheter, stent deployment began proximally and progressed distally as monitored under endoscopic and fluoroscopic guidance. After the deployment of the SEMS, the delivery system and guidewire were removed. Stent insertion was performed by 1 of 2 endoscopists (Sung-Bum Cho, Wan-Sik Lee). The 3 types of SEMS including Hanaro stent (M.I. Tech. Korea Co. Ltd., Seoul, Korea), Bona stent (Standard Sci-Tech Inc., Seoul, Korea), and Niti-s stent (Taewoong Medical Co., Seoul, Korea) were used. According to the endoscopist's preference and experience, stent type was chosen. Stent length was decided by allowing for more than 2 cm away from the distal and proximal margin of the obstructing lesion using fluoroscopy. The diameter and length of stent used were 20–25 mm and 6–12 cm, respectively. Immediately, 1 day, and 3 days after stent insertion, the patient underwent abdominal X-ray to access the position and location of stent. All the patients were observed during hospital stay to assess the technical, clinical success, and presence of complications. After their discharge, the patients were seen at follow-up at our institution until loss to follow-up.

### 2.3. Definitions and Data Analysis

Post-SEMS fever was defined as an increase in body temperature over 38.0°C within 7 days after the SEMS insertion. Post-SEMS SIRS was defined as two or more of following clinical manifestations after the SEMS insertion: (1) body temperature greater than 38°C or less than 36°C; (2) heart rate greater than 90 beats/minute; (3) respiratory rate greater than 20 breaths/minute or hyperventilation with an arterial partial pressure of carbon dioxide less than 32 mmHg; (4) white blood cell count (WBC) > 12000/mm^3^, <4000/mm^3^, or with >10% immature neutrophils [14]. Post-SEMS sepsis was defined as SIRS in response to an infectious process after the SEMS insertion [15]. Technical success was defined as a successful stent insertion across the whole length of the colon obstruction [16–18]. Clinical success was defined as the regression of obstructive symptoms (abdominal pain, vomiting, abdominal distension, and the inability to pass any stool or gas) within 48 hours after technical successful SEMS insertion [16–18]. The degree of obstruction was divided into two groups such as total or subtotal obstruction as described previously [16]. Subtotal obstruction was defined as a state with narrow stool caliber or the ability to pass only small amounts of liquid stool or gas, and total obstruction was decreased or absent bowel sounds or the inability to pass any stool or gas. The following variables were analyzed to compare between prophylactic antibiotics (PA) group and non-PA group: age, sex, body mass index, laboratory findings on admission, aims for stent insertion (preoperative versus palliative), degree of obstruction (total versus subtotal), presence of abdominal pain and abdominal tenderness, stent type (covered versus uncovered), stent length (<10 cm versus ≥10 cm), stent diameter (≤22 mm versus >22 mm), obstruction site, presence of mechanical ileus, presence of carcinomatosis peritonei, technical success, clinical success, and complications after SEMS insertion.

### 2.4. Endpoints

The primary endpoint was the comparison of the infectious complications after SEMS insertion in PA and non-PA groups. The secondary endpoint was the comparison of technical and clinical outcomes after SEMS insertion in PA and non-PA groups.

### 2.5. Statistical Analysis

All values are expressed as the means ± standard deviation (SD) and were analyzed by Student's *t*-test. The categorical variables were analyzed by the *χ* ^2^ test and Fishers exact test. Univariate and multivariate Cox proportional hazards models were used to assess risk factors for post-SEMS infectious complications and to compute hazard ratios and their 95% confidence intervals. We made a propensity score using a logistic model. All variables that differed significantly when comparing 2 groups were included in the logistic model, with backward selection. The propensity score was then used to adjust for efficacy of PA on post-SEMS infectious complications in a multivariable Cox model. The Statistical Package for the Social Sciences (SPSS/PC+ 18.0, Chicago, IL, USA) was used for all analyses. A value of *P* < 0.05 was accepted as statistical significance.

## 3. Results

### 3.1. Baseline Characteristics of Patient

A total of 224 patients were enrolled in this study. 145 patients received PA before the insertion of SEMS (PA group) and 79 patients did not receive PA (non-PA group) (Figure 1). The baseline characteristics of the patients in both groups are summarized in Table 1. Of these, 126 patients received SEMS as bridge therapy before curative surgery and 98 patients received SEMS as palliation in advanced disease. The mean CRP value of PA group was 4.6 ± 6.6, which was significantly higher than that of non-PA (*P* = 0.001). Symptoms or signs of complete obstruction such as abdominal tenderness and mechanical ileus were significantly more frequent in PA group than those in non-PA (*P* = 0.010 and *P* < 0.001, resp.). The most common location of the obstruction in both groups was the rectosigmoid colon (80.0% in PA group, 78.5% in non-PA group). The distribution of the obstruction locations was similar in both groups. The degree of colon obstruction was not significantly different between PA and non-PA groups (*P* = 0.530).

### 3.2. Technical and Clinical Outcomes

The technical and clinical outcomes after SEMS insertion in both groups are summarized in Table 2. Technical success rate was 95.1% (213/224) and was similar in both groups (95.9% (139/145) in PA group, 93.7% (74/79) in non-PA group, *P* = 0.524). Clinical success rate was 96.9% (217/224) and was also similar in both groups (97.9% (142/145) in PA group, 94.9% (75/79) in non-PA group, *P* = 0.240). Complications as a result of stent insertion, including perforation, migration, and reobstruction, were similar in both groups (*P* > 1.000, *P* = 0.826, and *P* = 0.807, resp.). Post-SEMS insertion fever and SIRS were developed in 15 (6.7%) and 24 (10.7%), respectively. Blood culture was performed in 137 patients. Among them, post-SEMS insertion bacteremia was developed in 8 (5.8%). All of fever, SIRS, and bacteremia occurred within 72 hours after SEMS insertion. The frequency of post-SEMS insertion fever, SIRS, and bacteremia was not significantly different between the PA and non-PA groups (*P* = 0.781, 0.174, and 0.696, resp.). There was no post-SEMS insertion sepsis in both groups. The time interval between symptom onset and SEMS insertion was not significantly different between the PA and non-PA groups (*P* = 0.529).

### 3.3. Risk Factors for Infectious Complications after SEMS Insertion

We used multivariate logistic regression analysis adjusted with PA, CRP, abdominal tenderness, and mechanical ileus as covariates to validate the independent risk factors related to post-SEMS insertion fever, SIRS and bacteremia. The CRP level was the risk factor related to post-SEMS insertion SIRS. However, any of these factors was not risk factor related to post-SEMS insertion fever and bacteremia (Table 3). As shown in Table 1, higher CRP level, abdominal tenderness, and mechanical ileus were significantly associated with patients received antibiotics before the insertion of SEMS. It means that both groups differ in some respects. Therefore, we built the propensity score-matched pairs between 2 groups to limit its selection bias. The propensity score included CRP, abdominal tenderness, and mechanical ileus. After propensity score matching, a total of 102 patients, 51 in the PA group and 51 in the non-PA group, were matched, and there was no significant difference in outcome of propensity score-matched post-SEMS insertion fever, SIRS, and bacteremia between 2 groups (Table 4).

### 3.4. Endpoints

*Primary Endpoint.* The frequency and risk factors of infectious complications (using a propensity score) after SEMS insertion were not significantly different between PA and non-PA groups. 

*Secondary Endpoint*. The technical and clinical outcomes after SEMS insertion were similar in PA and non-PA groups.

## 4. Discussion

Acute luminal obstruction is one of the common presentations of colorectal cancer [2, 3, 19]. Emergency surgery is the traditional treatment of choice but is associated with high morbidity and mortality [2]. Colon SEMS are well recognized and commonly used for preoperative decompression and palliation in malignant colorectal obstruction. Numerous studies have demonstrated that colon SEMS insertion is safe and effective on short-term basis compared with surgical interventions [6, 11, 20–22]. According to the recent published clinical guideline, SEMS insertion in malignant colorectal obstruction is recommended for the patients with symptomatic and radiological evidence, as an alternative to emergency surgery and as a palliative treatment. However, SEMS insertion is not recommended as a bridge to elective surgery and prophylactic treatment [7, 8].

The variety of endoscopic procedures is generally considered a low risk for development of infectious complications [23–25]. Despite the low risk of development of infectious complications, complications can be fatal, and for this reason, many clinicians administer pre- or perioperative antibiotics to patients undergoing high-risk endoscopic procedures including esophageal dilation, variceal sclerotherapy, and endoscopic retrograde cholangiopancreatogram with biliary obstruction [26–28]. Colon SEMS insertion is expected to have a higher risk of infectious complications than diagnostic colonoscopy because of the increase of wall tension and intestinal barrier damage causing bacterial translocation during the procedure and after [13, 29]. Therefore, colon SEMS insertion-related infection may occur under the following circumstances; use of contaminated equipment and accessories or spread of intestinal flora to bloodstream and adjacent tissues by mucosal injury as a result of the procedure. The prevention of infectious complications is the reason why many clinicians routinely prescribe a PA before stent insertion, especially the patients with total colonic obstruction [9]. In our study, many clinicians showed a tendency to prescribe a PA, if the patients had any symptoms or signs suggesting colon cancer obstruction, such as abdominal distension, abdominal tenderness, and presence of mechanical ileus and elevated CRP.

However, until now, little is known about the clinical impact of PA for reducing the infectious complications after stent insertion. The first aim of this study was whether the use of PA is effective to reduce the infectious complications after stent insertion. Contrary to expectations, the frequency of colon SEMS insertion-related fever, SIRS, and bacteremia was not significantly different between the PA and non-PA groups in our study. Also, previously, colorectal SEMS insertion was associated with a low rate of bacteremia, similar to that of diagnostic colonoscopy [13]. The next aim was to determine the potential risk factors of colon SEMS insertion-related fever, SIRS, and bacteremia. In multivariate analysis, the CRP level was only risk factor related to colon SEMS insertion-related SIRS, except for fever and bacteremia. However, patients of PA group showed higher CRP level, higher incidence of abdominal tenderness, and mechanical ileus than those of non-PA group. The propensity score included CRP, abdominal tenderness, and mechanical ileus. In propensity score matching analysis, there was no independent risk factor related to colon SEMS insertion-related fever, SIRS, and bacteremia. These results suggest that PA has limited value, if any, for reducing the incidence of infectious complications, and that the treatment of PA for SEMS insertion may be an unnecessary or immoderate treatment.

The inappropriate use of antibiotics is associated with an alteration in intestinal microflora in humans. This may result in ranging from mild diarrhea to* Clostridium difficile*-associated colitis-induced fever, abdominal pain, abdominal distention, leukocytosis, and life-threatening fulminant colitis causing hemorrhage and necrosis. Also, in light of current concerns regarding the development of antibiotics resistance and the lack of literature to support the decision of the clinicians to use antibiotics, it may be acceptable that patients undergoing colon SEMS insertion are not administered any pre-, peri-, or post procedure antibiotics treatment unless subsequent complications indicate that this is necessary.

However, our study has some limitations. First, the study design was retrospective and nonrandomized and selection biases were unavoidable. Thus, the propensity score matching analysis was used to reduce selection bias in our study. However, it remains the major concern of our study. Second, it was inevitable that the PA group was heterogenous. For these reasons, a large prospective, multicenter, randomized control trial evaluating the efficacy of PA in reducing the infectious complications after SEMS insertion for malignant colorectal obstruction is required to provide more definitive evidence. In conclusion, based on our retrospective study, the use of PA for colonic SEMS insertion in patients with malignant colorectal cancer obstruction has little or no influence on the prevention of febrile event and infectious complications.

## Figures and Tables

**Table 1:** Comparison of clinical characteristics in PA versus non-PA groups.

Characteristics	PA group (*N* = 145)	Non-PA group (*N* = 79)	*P* value
Age, years (mean ± SD)	67.6 ± 11.7	65.0 ± 12.6	0.128
Sex (male/female)	94 (64.8%)/51 (35.2%)	54 (68.4%)/25 (31.6%)	0.659
Body mass index (kg/m^2^)	21.6 ± 2.7	21.8 ± 2.7	0.538
Aims (preoperative/palliative)	82 (56.6%)/63 (43.4%)	44 (55.7%)/35 (44.3%)	1.000
Diabetes mellitus	21 (14.5%)	11 (13.9%)	1.000
WBC (×1000), (/mm^3^)	8.1 ± 3.2	7.5 ± 2.7	0.198
ANC (×1000), (/mm^3^)	5.8 ± 3.0	5.2 ± 2.5	0.124
CRP^∗^ (mg/dL)	4.6 ± 6.6	2.4 ± 3.4	0.001
Potassium (mEq/L)	3.9 ± 0.5	3.9 ± 0.8	0.556
Abdominal pain	112 (77.2%)	54 (68.4%)	0.154
Abdominal tenderness^∗^	66 (45.5%)	22 (27.8%)	0.010
Degree of obstruction (total/subtotal)	42 (29.0%)/103 (71.0%)	19 (24.1%)/60 (75.9%)	0.530
Mechanical ileus^∗^	80 (55.2%)	24 (27.8%)	<0.001
Carcinomatosis peritonei	18 (12.4%)	7 (8.9%)	0.509
Obstruction site			0.857
Ascending colon	3 (2.1%)	2 (2.5%)	
Hepatic flexure	8 (5.5%)	5 (6.3%)	
Transverse colon	3 (2.1%)	0 (0.0%)	
Splenic flexure	7 (4.8%)	5 (6.3%)	
Descending colon	8 (5.5%)	5 (6.3%)	
Rectosigmoid colon	116 (80.0%)	62 (78.5%)	

PA, prophylactic antibiotics; *N*, number; WBC, white blood cell; ANC, absolute neutrophil count; CRP, C-reactive protein; ^∗^significantly different.

**Table 2:** Comparison of procedure-related outcomes in PA versus Non-PA groups.

Characteristics	PA group (*N* = 145)	Non-PA group (*N* = 79)	*P* value
Stent type (covered/uncovered)	62 (42.8%)/83 (57.2%)	29 (36.7%)/50 (63.3%)	0.397
Stent length (<10 cm/≥10 cm)	99 (68.3%)/46 (31.7%)	52 (65.8%)/27 (34.2%)	0.766
Stent diameter (≤22 mm/>22 mm)	63 (43.4%)/82 (56.6%)	28 (35.4%)/51 (64.6%)	0.244
Technical success	139 (95.9%)	74 (93.7%)	0.524
Clinical success	142 (97.9%)	75 (94.9%)	0.240
Complications			
Perforation	4 (2.8%)	2 (2.5%)	1.000
Migration	17 (11.7%)	8 (10.1%)	0.826
Reobstruction	13 (9.0%)	6 (7.6%)	0.807
Fever	9 (6.2%)	6 (7.6%)	0.781
SIRS	19 (13.1%)	5 (6.3%)	0.174
Bacteremia/blood culture	5/96 (5.2%)	3/41 (7.3%)	0.696
Time interval (hours) between symptom onset and stent insertion	232.8 ± 200.2	233.1 ± 150.5	0.529

WBC, white blood cell; ANC, absolute neutrophil count; CRP, C-reactive protein; PA, prophylactic antibiotic; SIRS, systemic inflammatory response.

**Table 3:** Multivariate analysis of factors associated with post-SEMS insertion fever, bacteremia, and SIRS.

Factors	Fever		Bacteremia		SIRS	
	OR (95% CI)	*P* value	OR (95% CI)	*P* value	OR (95% CI)	*P* value
Prophylactic antibiotics	1.55 (0.48–4.98)	0.459	1.79 (0.36–8.85)	0.477	0.67 (0.22–2.02)	0.478
CRP (mg/dL)	1.06 (0.99–1.14)	0.102	1.02 (0.92–1.13)	0.684	1.12 (1.05–1.19)	<0.001
Abdominal tenderness	0.48 (0.16–1.45)	0.194	0.59 (0.14–2.54)	0.477	0.86 (0.34–2.19)	0.758
Mechanical ileus	1.51 (0.48–4.71)	0.478	0.83 (0.19–3.69)	0.804	0.92 (0.36–2.35)	0.866

SEMS, self-expandable metallic stent; OR, odds ratio; CI, confidence interval; CRP, C-reactive protein; SIRS, systemic inflammatory response syndrome.

**Table 4:** Outcome of propensity score-matched post-SEMS insertion fever, SIRS, and bacteremia between PA and non-PA groups.

Characteristics	PA group (*N* = 51)	Non-PA group (*N* = 51)	Odds ratio (95% CI)	*P* value
Fever	2 (3.9%)	5 (9.8%)	0.376 (0.690–2.032)	0.436
Bacteremia	3 (5.9%)	1 (2.0%)	3.125 (0.314–31.094)	0.617
SIRS	7 (13.7%)	3 (5.9%)	2.545 (0.620–10.458)	0.318

SEMS, self-expandable metallic stent; PA, prophylactic antibiotic; OR, odds ratio; CI, confidence interval; SIRS, systemic inflammatory response syndrome.
